# Tracking Fecal Bacterial Dispersion from Municipal Wastewater to Peri-Urban Farms during Monsoon Rains in Hue City, Vietnam

**DOI:** 10.3390/ijerph18189580

**Published:** 2021-09-11

**Authors:** Windra Prayoga, Masateru Nishiyama, Susan Praise, Dung Viet Pham, Hieu Van Duong, Lieu Khac Pham, Loc Thi Thanh Dang, Toru Watanabe

**Affiliations:** 1 United Graduate School of Agricultural Sciences, Iwate University, 18-8 Ueda 3-Chome, Morioka 020-8850, Japan; windra.prayoga@gmail.com; 2 Department of Food, Life and Environmental Sciences, Faculty of Agriculture, Yamagata University, 1-23 Wakaba-Machi, Tsuruoka 997-8555, Japan; dphamviet1986@yahoo.co.jp (D.V.P.); to-ru@tds1.tr.yamagata-u.ac.jp (T.W.); 3 Faculty of Environmental Science, University of Sciences, Hue University, 77 Nguyen Hue St., Hue City 49100, Vietnam; dvhieu@hueuni.edu.vn (H.V.D.); pklieu@hueuni.edu.vn (L.K.P.); dangthithanhloc@hueuni.edu.vn (L.T.T.D.)

**Keywords:** *E. coli* contamination, agricultural farms, multilocus sequence typing, urban drainage, flooding

## Abstract

Disease outbreaks attributed to monsoon flood-induced pathogen exposure are frequently reported, especially in developing cities with poor sanitation. Contamination levels have been monitored in past studies, yet the sources, routes, and extents of contamination are not always clear. We evaluated pollution from municipal wastewater (MWW) discharge and investigated fecal contamination by *Escherichia coli* (*E. coli*) in three agricultural fields on the outskirts of Hue City, Vietnam. After *E. coli* concentration was determined in irrigation water (IRW), MWW, soil, vegetables (VEG), and manure, its dispersion from MWW was tracked using multilocus sequence typing (MLST) and phylogenetic analyses during the wet and dry seasons. IRW was severely contaminated; 94% of the samples were positive with *E. coli* exceeding the stipulated standards, while VEG contamination was very low in both seasons. The confirmed total number of isolates was comparable between the seasons; however, results from MLST and phylogenetic clustering revealed more links between the sites and samples to MWW during the wet season. The wet season had four mixed clusters of *E. coli* isolates from multiple locations and samples linked to MWW, while only one mixed cluster also linking MWW to IRW was observed during the dry season. The most prevalent sequence type (ST) complex 10 and two others (40 and 155) have been associated with disease outbreaks, while other STs have links to major pathotypes. Irrigation canals are significant routes for *E. coli* dispersion through direct links to the urban drainage-infested river. This study clarified the genotype of *E*. *coli* in Hue city, and the numerous links between the samples and sites revealed MWW discharge as the source of *E. coli* contamination that was enhanced by flooding.

## 1. Introduction

Extreme flooding is a major cause of weather-related infectious disease outbreaks with strong links to diarrheal diseases [1,2]. The frequency and intensity of extreme precipitation events are projected to increase [1,3,4]; thus, floods and the associated impacts will increase. Flooding overwhelms drainage systems and, in some cases, allows the mixing of waste from different sources [1,5]. Degraded water quality, waterborne disease, and infectious diarrhea remain prominent and persistent problems worldwide, and they are expected to worsen under future climate change projections [2]. Environmental pollution associated with floods leads to health problems as enteric pathogens are mobilized and dispersed to surrounding areas during flooding [3,6]. Various studies by de Man et al. [7], ten Veldhuis et al. [8], and Yu et al. [6] have revealed the presence of high numbers of both *Escherichia coli* and intestinal enterococci in floodwater [9].

Extreme rainfall events have been shown to trigger waterborne disease outbreaks via infrastructural inundation, hydrological short-circuiting/preferential flow, and the subsequent consumption of contaminated water [3]. Past studies revealed increased rates of diarrhea and other illnesses after heavy rainfalls and flooding, suggesting an increase in post-flooding microbial loads, yet the individual pathogens present in the water [9] and the extent of microbial dissemination have not been explored. Further, floodwater can contaminate widely across flooded areas, including the sources of domestic water supplies and agricultural fields, and the pathogens carried in floodwater can persist for long periods after flooding has ceased [10,11,12], including up to 238 days in agricultural soils [13]. The flooding of agricultural fields is a known source of contamination, though microbiological contamination in fields has been infrequently measured [10,13]. The lack of data on the levels, extents, and spatial patterns of contamination, which can vary greatly, hinders remediation efforts. Such information is important for understanding the remediation time required to reduce contamination to safe levels [14]. Regardless of the route of transmission, exposure to pathogens in floodwater poses a severe public health risk and contributes to the global disease burden and preventable mortality [11,15].

Southeast Asia is prone to flooding due to seasonal rains induced by the passage of the East Asian Monsoon. Out of 161 extreme flood disasters reported worldwide in 2016, 43% occurred in Asia [16]. Vietnam is one of the most flood-prone countries, and coastal Hue City is among the cities most vulnerable to seasonal flooding from monsoon rains [17]. Hue city features a tropical monsoon climate with high temperatures, plentiful radiation, and a distinctive rainfall regime [18]. It is one of the areas that receive the highest rainfall in Vietnam: heavy rainfall occurs from October to December, with an average precipitation of ~2682 mm per year (climate data available online: http://en.climate-data.org (accessed on 14 February 2021)). Hue City and several other cities in Vietnam were struck by floods in December of 2018 (EM-DAT, available online: http://emdat.be (accessed on 28 January 2020). When flooding occurs, floodwaters flow directly downstream along the Perfume River, likely carrying contaminants to the lower floodplain. In Vietnam, approximately 92% of the urban wastewater collection is conducted via combined sewerage and drainage systems, collecting stormwater and wastewater via pipeline networks. Like many cities in Vietnam, Hue city does not have a WWTP; instead, wastewater from homesteads is mainly pretreated in household septic tanks combined with the toilet effluent before being discharged into sewer systems and then into water bodies [18]. The contaminated water is withdrawn for irrigation through irrigation canals that connect directly to the water bodies, specifically the Perfume River in Hue city, which receives the most sewer drainage.

Understanding the impacts of different extreme water-related weather events on waterborne diseases is an essential step toward mitigating the associated risks [15]. Therefore, assessing the level and extent of contamination under changing weather conditions is crucial. The monitoring of indicator organisms is a common approach used to quantify pathogen contamination loads. However, with microbial source tracking, it is also possible to trace the spread and assess the extent of contamination [19]. *E. coli* is a ubiquitous commensal in the human gastrointestinal tract [20]. Usually, the commensal *E. coli* and its host can co-exist mutually benefiting one another; however, several highly adapted *E. coli* clones have acquired specific virulence traits that allow them to adapt to new niches and cause a broad spectrum of disease in their hosts [21,22]. Opportunistic pathogenic *E. coli* can cause a range of diseases based on the strains, which are classified into pathotypes [21,22,23]. All pathotypes have an enormous potential to cause diseases, including diarrheal, which is a serious public health concern because of its morbidity and mortality in children and global outbreaks [23]. The major pathotypes are: (i) extra-intestinal pathogenic *E. coli* (ExPEC), which is associated with neonatal meningitis (NMEC), and urinary tract infections caused by uropathogenic *E. coli* (UPEC), and also includes the bird and avian pathogenic *E. coli* (APEC); (ii) diarrhea and intestinal pathogenic *E. coli* (InPEC), which is associated with diarrheal diseases and is sub-divided into enteropathogenic *E. coli* (EPEC), enterohemorrhagic *E.coli* [EHEC] (e.g., Shiga toxin-producing *E. coli* (STEC)), *Shigella*/enteroinvasive *E. coli* (EIEC), enteroaggregative *E. coli* (EAEC), diffusely adherent *E. coli* (DAEC), enterotoxigenic *E. coli* (ETEC), and adherent invasive *E. coli* (AIEC) [21,22,23].

Contamination with fecal bacterial and protozoan parasites has been reported in vegetables on sale in Hue City [24,25]. All vegetable samples examined by Ho et al. [25] were highly contaminated with aerobic bacteria and *E. coli*, ranging from 6.84–8.40 log CFU/g and 5.47–6.88 log CFU/g, respectively [25]. High foodborne illness and food poisoning occurrences in Vietnam have raised concerns about food safety, especially in the vegetable sector [26]. Most recently, *Salmonella* with antibiotic resistance traits was isolated from vegetables on retail in a neighboring city [27]. Unfortunately, the sources, levels, and spread of pathogens in Hue City, Vietnam, have never been explored, yet this area experiences several floods every year. The partially treated wastewater from urban discharge and frequent flooding leads to fecal bacterial contamination in the urban area and the outskirts including agricultural fields. Agricultural areas are at a high risk of contamination from floods and irrigation water drawn from the Perfume River. The aim of this study was to trace and identify the source of microbial contamination and examine the impact of flooding on microbial spreading to peri-urban agricultural farms. We investigated bacterial contamination in agricultural fields located on the outskirts of Hue City using *E. coli* as the indicator organisms to determine the level of contamination during the dry and wet seasons. We further applied multilocus sequence typing (MLST) to examine the links between the contaminated agricultural sites, samples, and urban drainage—the suspected contaminant source. Our findings are expected to provide insights into the contamination of peri-urban farmlands, which serve as vital sources of food and economic development and are also key in foodborne disease outbreaks in the region.

## 2. Materials and Methods

### 2.1. Sampling

**Figure 1:**
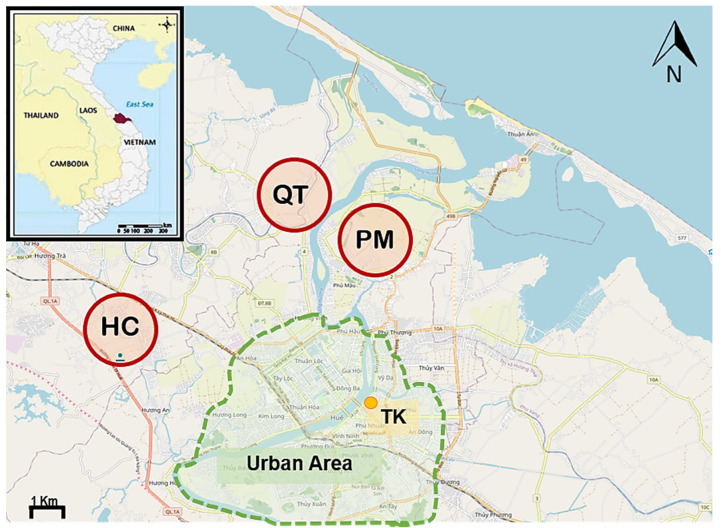
Location of sampling in Hue, Vietnam. Irrigation water (IRW), soil (SOL), and manure (MNR) samples were taken from Huong Chu (HC), Phu Mau (PM), and Quang Thanh (QT) communes in red circles. Municipal wastewater (MWW) samples were collected from the drainage pipe at Toa Kham (TK) (orange dot). The light-green shaded area shows extent of the city; however, only the northeast section drainage flows through TK.

This study was conducted from May of 2018 to April of 2019. A total of 331 samples, consisting of 2 municipal wastewater (MWW), 121 irrigation water (IRW), 68 vegetables (VEG), 142 soil (SOL), and 2 manure (MNR) samples, were collected from Thien Hue province in central Vietnam for *E. coli* enumeration during monthly campaigns. The samples were grouped into two seasons—dry and wet—and the designated months were used as seasonal representatives (i.e., June and July for the dry season and November and December for the wet season) for source tracking. During the sampling period, total precipitation in the study area was 2304.8 mm with the highest amount received in December (745.1 mm) [28]. The onset of flooding usually occurs with heavy rainfall (>700 mm); however, the cumulative precipitation together with other factors such as the duration will induce downstream flooding. The total amount of rainfall received during the wet season was 6.5 times higher than in the dry season. Municipal wastewater samples were taken from a wastewater drainage pipe in the Toa Kham (TK) area of Hue City, while IRW, SOL, VEG, and MNR samples were collected from agricultural areas in three communes—Huong Chu (HC), Phu Mau (PM), and Quang Thanh (QT)—surrounding the city (Figure 1).

The farms were selected based on: (1) the location, i.e., in the upstream not influenced by MWW (HC), downstream with MWW influence close and far away from the city (PM and QT, respectively), (2) source of IRW, (3) farmer’s permission, and (4) the type of vegetables grown. We selected lettuce, which is the most grown vegetable in Hue province, for our study. Lettuce is grown all year round except during low temperatures (<15 °C) and heavy floods. Mature leaves which were ready for harvest at the time of sampling were picked from the plant by hand (during the growing season) and stored in polyethylene bags before transporting to the laboratory on ice. Soil samples (0–10 cm) were collected using a sterile scope from three sampling points in one lettuce row and thoroughly mixed to make a composite for analysis. After sampling, all samples were transported to the laboratory on ice in a cooler box and microbial analysis was started immediately i.e., within 6 h of sample collection.

In each commune, five SOL and VEG sampling sites were selected, including one upstream of the IRW inlet, which was designated as a control for SOL samples. Meanwhile, IRW sites were three, four, and one from PM, QT, and HC, respectively, based on the VEG samples and the supply canal. Manure was only collected from two sites at PM and QT since it was not applied in HC. During the growing season, farmers sometimes use manure that is either commercially composted or prepared locally by mixing chicken and pig muck, cow dung, rice straw, and husks. The mixture is then piled and covered with canvas and left to compost for about 3–4 months without probiotics. If probiotics, mainly *Trichoderma* or *Bacillus* sp, are used, the total composting time reduces to 45 days.

### 2.2. Counting and Isolation of E. coli

*E. coli* was isolated on a Chromocult^®^ Coliform Agar medium (Merck & Co., Inc., Darmstadt, Germany). One mL of MWW and IRW samples were separately taken and diluted with 9 mL sterile saline (0.85% NaCl, total volume: 10 mL) to obtained dilution up to 10^−2^. Next, 100 μL of MWW and IRW samples (original, 10^−1^, and 10^−2^ diluted) were poured directly onto the surface of the medium. For solids (SOL and MNR) and VEG samples, 1 and 20 g were rinsed with 9 and 100 mL of sterile saline, respectively [29]. 100 μL of the rinsed mixtures were then poured and spread on the aforementioned media. The inoculated media were incubated at 37 ºC for 24 h, and the blue colonies suspected to be *E. coli* were counted and isolated for further testing. The number of *E. coli* for each of the samples was determined from the mean colony forming units (CFU) on three replicate Chromocult^®^ Coliform Agar media. *E. coli* for seasonal representative months (June and July for the dry season and November and December for wet season) were processed for further analysis

### 2.3. Identification of E. coli Isolates

Suspected *E. coli* isolates were confirmed by detecting the *uidA* gene via polymerase chain reaction (PCR) [30]. The DNA of suspected isolates was extracted using the InstaGene Matrix (Bio-Rad Laboratories, Inc., Hercules, CA, USA) and used as the DNA template. Polymerase chain reaction analyses were conducted using the KAPA Taq Extra PCR Kit (KAPA Biosystems, Inc., Boston, MA, USA) and a primer set consisting of the forward primer uidA-F (5′TGGTAATTACCGACGAAAACGGC3′) and reverse primer uidA-R (5′ACGCGTGGTTACAGTCTTGCG3′). The PCR conditions were as follows: three minutes at 95 °C for initial denaturation, 35 cycles of 30 s each at 95 °C for denaturation, 30 s at 58 °C, and one minute at 72 °C for elongation, followed by ten minutes at 72 °C for final elongation. The presence of a 162-base pair (bp) band was confirmed via gel electrophoresis of the PCR products, indicating the presence of the *uidA* gene.

### 2.4. Multilocus Sequence Typing

As a genetic-based tracing method, multilocus sequence typing (MLST) analyses using the Achtman scheme were employed to the confirmed *E. coli* isolates [31]. MLST identifies the internal nucleotide sequences of multiple locus-encoding housekeeping genes, or fragments of them, where unique sequences are assigned a random integer number and a unique combination of alleles at each locus (or “allelic profile”), specifying the sequence type (ST) [32,33]. Whole-genome sequencing has higher discrimination power for comparing allelic profiles; however, MLST can attain similar levels of discrimination with fewer loci using just seven housekeeping genes [32]. Furthermore, MLST can identify and track the global spread of pathogens [34] and is, therefore, beneficial and recommended for assessing microbial contamination.

Seven *E. coli* housekeeping genes (*adk*, *fumC*, *gyrB*, *icd*, *mdh*, *purA*, and *recA*) were individually amplified by PCR using the KAPA Taq Extra PCR Kit. The PCR conditions were set as two minutes at 95 °C for initial denaturation, 30 cycles of one minute at 95 °C for denaturation, two minutes at an annealing temperature for each primer set, as shown in Table 1, two minutes at 72 °C for elongation, and five minutes at 72 °C for final elongation. Overall, we used MLST standard protocols [31], including the primers for the housekeeping genes, which were adopted with modifications at the annealing temperatures for some primers to minimize the primer–dimer and the undesired non-target amplification.

The PCR products were analyzed with 2% agarose gel electrophoresis, followed by purification using ExoSAP-IT™ PCR Product Cleanup (Applied Biosystems, Foster City, CA, USA). Sequencing was performed at FASMAC Co., Ltd. (Atsugi, Kanagawa, Japan) using ABI Genetic Analyzer 3130XL or ABI DNA Analyzer 3730xL (Applied Biosystems, Foster City, Japan) and BigDye Terminator v3.1 Cycle Sequencing Kits (Applied Biosystems). Primers shown in Table 1 were used for sequencing, and the sequences of each housekeeping gene were read from both ends. Sequence chromatographs were edited with a Sequencher^TM^ software, ver. 5.4.6 (Gene Code Corporation, Ann Arbor, MI, USA). The data of each of the housekeeping genes sequenced from both ends were assembled. The consensus sequence of housekeeping genes, with specific lengths for each allele, were plotted into the MLST database at Enterobase to identify each allele [35]. The sequence type (ST) and ST complex (STc) of each *E. coli* isolate were determined from the combination of the seven allelic identities. The Enterobase database was further checked for the phylogroup of all isolates.

### 2.5. GrapeTree Analysis

Seven allelic profiles of each isolate obtained from Enterobase were also used in the GrapeTree analysis to visualize the relationship among STs of each isolate in dry and wet seasons. These allelic profiles together with their metadata, which include information of STs and the sample type of isolates, were analyzed using GrapeTree software with the novel minimum spanning tree (MSTreeV2) method to construct the GrapeTree [36].

### 2.6. Phylogenetic and Diversity Analysis

Phylogenetic and nucleotide diversity using the seven alleles combined for MLST (*adk*–*fumC*–*gyrB*–*icd*–*mdh*–*purA*–*recA*) were performed to determine the relatedness of each *E. coli* strain using MEGA X software [37]. Phylogenetic trees were constructed via neighbor-joining using the Kimura-2 parameter algorithm, while the bootstrap method with 500 replications was used for nucleotide diversity. The resulting clusters were identified and the pathogenicity of isolates forming the clusters was then identified from the Enterobase if available. These analyses were applied to the isolates of *E. coli* collected during the dry and wet seasons separately to compare the spread of each *E. coli* strain in the study area between the two seasons.

### 2.7. Data Analysis

Wilcoxon signed-rank tests were performed to identify any significant differences in the *E. coli* concentrations of VEG, SOL, and IRW samples between the dry and wet seasons. Statistical analyses were conducted using R v. 3.6.3 [38] at an α-level of *p* ≤ 0.05. The results were compared to the Vietnamese standards [39,40] and to the United States Food and Drug Administration regulations [41] to evaluate the level of contamination in IRW, VEG, SOL, and MNR samples.

## 3. Results

### 3.1. Concentration of E. coli

The highest concentration of *E. coli* was noted in the MWW sample from TK at 39,500 CFU/mL or 4.6 log_10_ CFU/mL and 1344 CFU/mL or 3.1 log_10_ CFU/mL during the dry and wet season, respectively (Table S1). The MNR representative samples collected from the compositing area during the wet season from PM and QT had *E. coli* counts of 1585 CFU/g or 3.2 log_10_ CFU/g and 13 CFU/g or 1.1 log_10_ CFU/g, respectively (Table S1).

**Figure 2:**
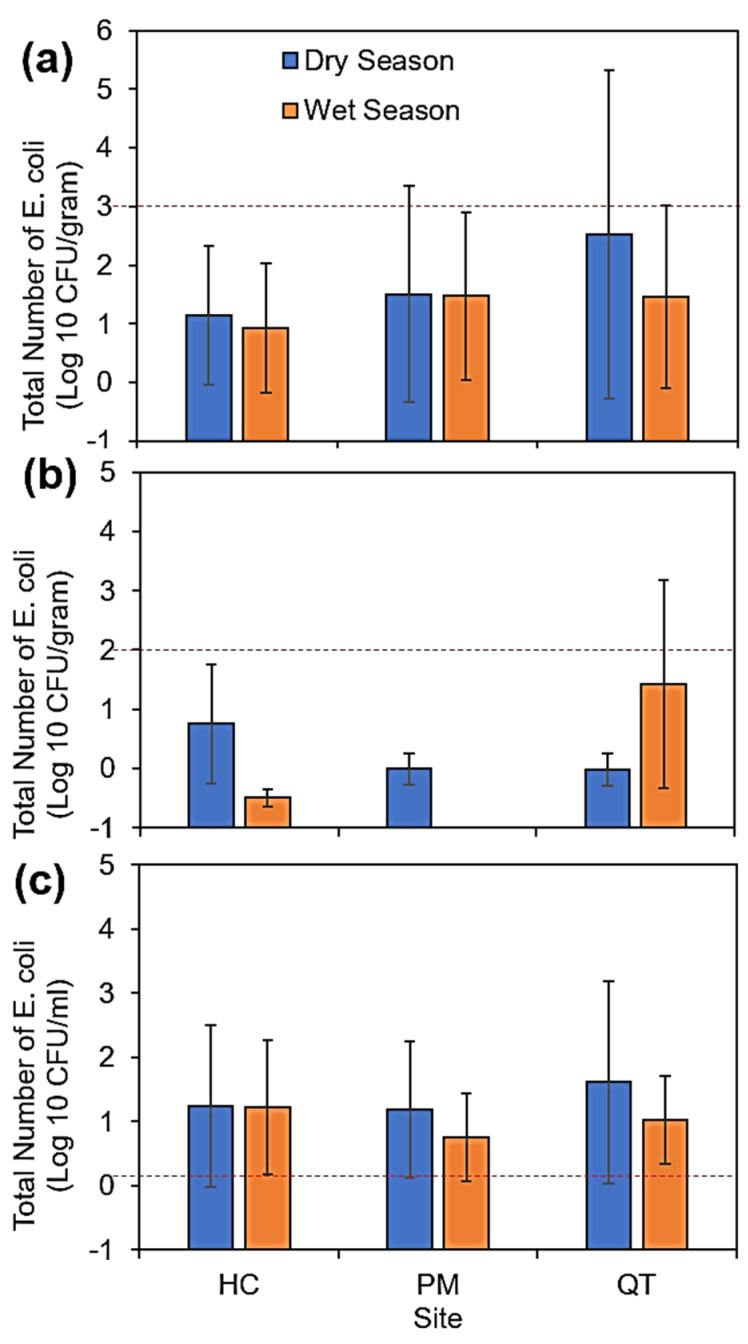
Mean concentrations of *E. coli* in (**a**) SOL, (**b**) VEG, and (**c**) IRW samples from Huong Chu (HC), Phu Mau (PM), and Quang Thanh (QT) communes. The dotted lines show recommended standards for SOL (US −FDA, 2020) and VEG and IRW (Vietnamese standards) (Ministry of Health, 2012; Ministry of Natural Resources and Environment, 2015). Statistical analysis using Wilcoxon signed-rank test did not show significant differences between the sites and seasons (*p* > 0.05).

As shown in Figure 2, higher concentrations of *E. coli* were found in SOL and IRW samples during the dry season. The counts showed extreme monthly fluctuations, especially in VEG samples, as indicated by the high standard deviation. The seasonally averaged bacterial counts for VEG, SOL, and IRW samples are shown in Figure 2. In the dry season, high *E. coli* were recorded for both SOL and IRW samples at all sites and for VEG samples at the HC and PM sites (Figure S1). The IRW was highly contaminated, with the highest frequency of detection among all samples; 97.2% (*n* = 35/36) *E. coli* positive samples, of which 94.3% (*n* = 33/35) had concentrations exceeding 2.0 CFU/mL—the maximum permissible limit under Vietnamese [39]. A small proportion of VEG samples (22%, 8/36) were positive for *E. coli*, but the level was within the acceptable limit of 2 log_10_ CFU/g according to national regulations for inspecting vegetables on the market [40]. However, the *E. coli* counts in VEG from QT were higher during the wet season than those from HC and PM. Among SOL samples, 69.4% (*n* = 25/36) were positive for *E. coli*, and only one sample had a concentration exceeding the US−FDA stipulated standard of 1000 MPN (3 log_10_ CFU/g [41]. It should be noted that this soil standard (US−FDA) [41] is based on the most probable number method rather than the total plate count method, with the colony-forming unit used in this study. Despite the detection frequency, no significant differences were observed among samples or the two seasons (Figure 2). Moreover, seasonal peak month observations (December (wet) and June (dry)) also did not reveal any significant differences.

### 3.2. E. coli Isolates

A total of 177 isolates collected from four sampling locations were confirmed as *E. coli*. Ninety isolates (50.9%) were collected during the dry season, and 87 (49.1%) were collected during the wet season (Table 2).

### 3.3. Sequence Type Identities of E. coli Isolates

**Figure 3:**
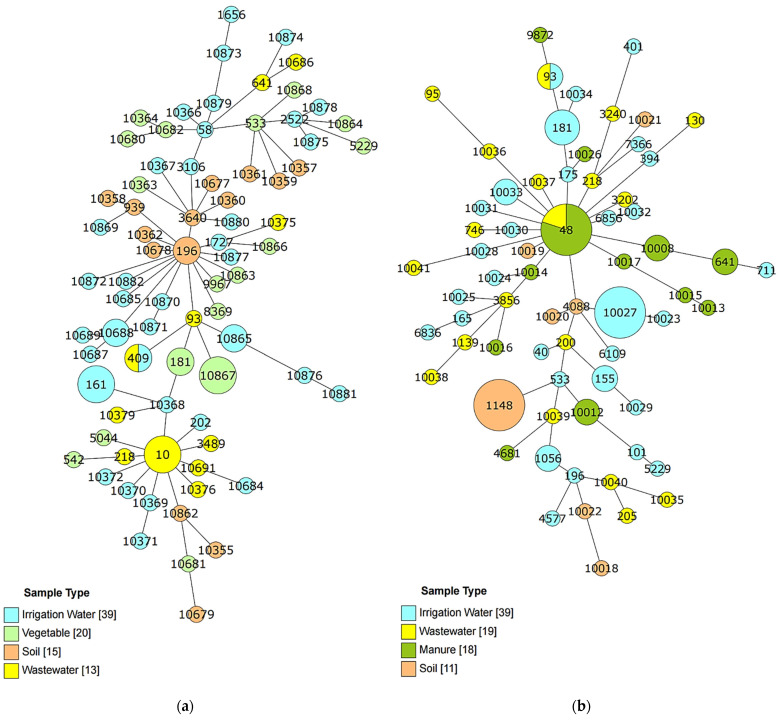
GrapeTree phylogeny constructed using sequence types (STs) identified by multilocus sequence typing during (**a**) the dry season and (**b**) the wet season and their relation in different samples. Each node represents a single ST, the number is the ST identity. The size of the node relates to the number of the isolates for each ST, i.e., the more the isolates with the same ST the bigger the size, and the color shows the sample type.

Multilocus sequence typing identified a total of 138 unique STs from the 177 confirmed *E. coli* isolates (Figure 3); 75.1% were singletons, while the remaining 24.9% were associated with STcs (Table S2). The STs were highly diverse with only 5.1% (7/138) of the unique STs shared between the two seasons.

In the dry season, the 90 confirmed *E. coli* isolates comprised seventy-nine STs, of which eight (ST10, ST161, ST10867, ST181, ST196, ST409, ST10865, and ST10688) were shared among two or more isolates (Figure 3a, Table S2). The predominant STs were ST10, ST161, and ST10867, which were found in three isolates from MWW, IRW, and VEG samples, respectively. Notably, all STs, except for ST409, which was present in both MWW (TK) and IRW (QT), were localized to a single sample type in a specific commune (i.e., ST181 was detected twice in VEG from HC, ST196 was identified in two SOL isolates from QT, and ST10865 and ST10688 strains were exclusive to IRW isolates from QT and PM, respectively).

Only 66 STs were identified from the wet season isolates (Table S2), 11 of which were shared among 32 isolates, and 43.9% of these STs had a corresponding STc dominated by STc 10. The most common STs among the isolates, ST48, ST1148, and ST10027, were detected in IRW at PM (ST10027), SOL (ST1148) from PM, MNR (ST48) from PM and QT, and in MWW. Similar to ST48, ST93 was also found in two isolates (IRW from PM and MWW), while ST155 was identified in the IRW of two communes, HC and QT. The most widespread isolates (ST48–STc10) were identified in MWW from TK, two IRW samples and MNR from QT, and IRW from PM. All isolates with shared STs included MWW isolates, indicating the transport of *E. coli* via wastewater. These isolates included: ST93 (MWW and IRW), ST181–STc 181 (two VEG samples in the dry season and three IRW samples from HC), ST53–STc 40 (VEG samples in the dry season from HC and IRW in the wet season from PM), ST641–STc 86 (MWW in the dry season and two MNR samples in the wet season), and ST5229–STc 101 (VEG samples from HC and IRW from QT during the wet season).

### 3.4. Relatedness of E. coli Isolates

**Figure 4:**
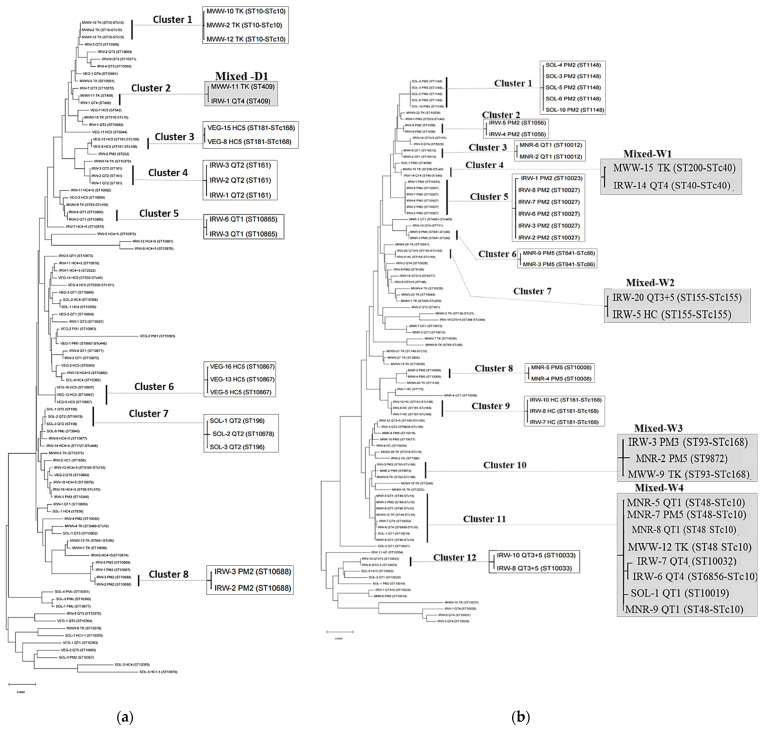
Phylogenetic tree constructed using the neighbor-joining tree method with Kimura 2-parameter model for *E. coli* isolates from (**a**) municipal wastewater (MWW), irrigation water (IRW), soil (SOL), and vegetable (VEG) samples collected during the dry season; (**b**) MWW, IRW, SOL, and manure (MNR) samples collected during the wet season taken from Huong Chu (HC), Phu Mau (PM), and Quang Thanh (QT) communes. Mixed clusters are highlighted in grey. For the two unrooted trees we constructed using MEGA-X software, the scale bars show a 5% difference between sequences aimed at finding the relationship among isolates without any need to depict the common ancestor.

The results of the phylogenetic analyses are shown in Figure 4. The phylogenetic tree for the dry season showed eight clusters with only one mixed (cluster D1, Figure 4a) composed of MWW (TK) and IRW (QT) isolates. In contrast, twelve clusters with four mixed clusters were identified in the wet season (W1–W4, Figure 4b). The first mixed cluster (W1) consisted of isolates from MWW from TK and IRW from QT. Mixed cluster W2 was composed of two isolates from IRW collected from two different communes (HC and QT). The third mixed cluster (W3) had three isolates from MWW samples (TK), IRW and MNR samples from the PM sites, while the fourth and largest mixed cluster (W4) included IRW, SOL, and MNR isolates from two communes (QT and PM) and MWW from TK. The three mixed clusters (W1, W3, and W4) encompassed MWW isolates from TK, which was the suspected source of the *E. coli* strains in the studied farms. The only mixed cluster in the dry season (D1) showed resemblance to the wet season cluster (W2), which was also comprised two isolates from MWW at TK and IRW at QT (Figure 4). The results from nucleotide diversity did not show clear differences between the seasons (data not shown).

From the previous studies, only 42.9% (76/177) of the isolates in our study were classified as phylogroup from the Enterobase. The isolates were mainly classified into phylogroup A, comprising 37 isolates and B with 38 B1 isolates and one B2 (Table 3 and Table S2). The pathotypes indicated some STs associated with various disease outbreaks, and the predominant pathotypes were EAEC, UPEC, ExPEC, and APEC (Table 3). Although the available information in the database was not enough to make comprehensive comparisons, MWW had diverse *E. coli* from A, B1, B2, and E dominated with phylogroup A, while both A and B were equally abundant (IRW Table 3).

## 4. Discussion

The levels and seasonal variations in *E. coli* concentrations observed in this study reflect the source and extent of the contamination. The high loads above permissible limits in IRW result from the direct connection to the Perfume River, which receives partially treated MWW discharged from the city. The low traces of vegetable contamination, less than previously reported by Ho et al. [25], may be attributed to the sampling time and technique as well as the amount of precipitation. During sampling, only mature leaves were picked from the plant during the farming period, which varies from most of the previous studies that examined already harvested produce. The scale of contamination in any flood depends on several factors, including the extent and severity of flooding, sediment load in the floodwater, and other characteristics specific to the storm, waterway, soil, and farm management practices [14]. The heavy precipitation from monsoon rainfall comes with surface run-off and always triggers floods in and around Hue city. The highly contaminated urban drainage mixes with stormwater and run-off as the water level increases; the contaminants in river water and IRW are then dispersed into the agricultural farms. In addition, the harvested vegetables are always washed and sprayed with water from the closest available source (usually canal or river) to stay fresh for sale in the local markets [24]. This practice will transfer the contaminants from the water to the vegetables contributing to the high level of *E. coli* as observed in the previous study by Ho et al. [25].

The lower abundance of *E. coli* during the wet season in most samples was due to dilution by precipitation and floodwater since rainfall increases stormwater while flushing accumulated pathogens into surface waters or dispersing them to the surrounding environment through run-off [10]. Contamination related to urban drainage will therefore be widespread during the wet season regardless of the low *E. coli* concentrations. Although *E. coli* are enteric bacteria, some strains can survive and even regrow (multiply) outside of an animal host in secondary environments [10,42,43]. The status of the Perfume River and the IRW canals characterized by high nutrients loads from agricultural run-off and fish culturing activities downstream presents a conducive environment for *E. coli* growth and multiplication

We suspected partially treated wastewater from urban drainage as the source of fecal contamination. However, the high amount of *E. coli* in both MWW and MNR samples indicated that both sources could be the source of farm contamination. The high loads noted in VEG samples from HC and QT, regardless of the correspondingly low *E. coli* counts in SOL samples, along with the high detection frequency in IRW, points to MWW as the major source for *E. coli* dispersion, as these two sites had no or very low loads in their MNR samples. In addition, MNR samples were obtained from the composting site, which may not reflect the exact composition at the time of the application. At the time of manure application, *E. coli* levels are expected to have drastically decreased, as noted from the differences in the two MNR samples. The high variation in *E. coli* concentrations observed between the downstream area (PM), which directly connects to the IRW through the Perfume River and is closer to the city and the upstream site (HC), reinforced our assumption of MWW as the main source of contamination. In this study, site location, as well as the *E. coli* loads in soil, highlighted the relevance of urban drainage and flooding. We noted differences between SOL and VEG contamination levels among the sites, specifically high loads at QT during the wet season. The location and soil ecosystem are important since the soil ecosystems are shaped by several factors, such as the nature, acidity, and alkalinity of the soil, and water levels which influence the occurrence and dissemination of microbes/pathogens [44]. Areas downstream are prone to microbial contamination from temporal rainfall even when IRW is not used during the wet season. The long rainy season and extreme rainfall associated with the monsoon usually results in flooding. During low rainfall intensity and at the beginning of the wet season, excess water is easily removed via conventional drainage networks, but these get challenged when extreme rainfall events increase [45]. The overwhelmed drainage system, which links to the farms via irrigation canals and the Perfume River, overflows spreading the contaminants, hence the high loads in SOL at QT.

The ST identities of each *E. coli* strain in samples from both seasons revealed that the genetic diversity varied seasonally, with only a few STs being common between the seasons. The *E. coli* based on STs were more widespread in the wet season than in the dry season. The heavy rainfall received in Thua Thien Hue (November–December of 2018) resulted in the spread of *E. coli* discharged from wastewater. From the shared STs, it can be inferred that *E. coli* spread across the three communes to MNR, IRW, and SOL, as revealed by the wet season isolate clusters. Three mixed clusters containing MWW isolates from TK among MNR, IRW, and SOL isolates revealed their relatedness to MWW, which is believed to be the primary source of microbial contamination to the Perfume River. One cluster in the wet season (W2) showed similarities with mixed cluster D1, as opposed to the other three clusters (W1, W3, and W4), and clusters W1, W3, and W4 likely resulted from intensified rainfall carrying contaminants in floodwaters downstream to the QT and PM communes. One key route for spreading bacteria besides flooding is IRW [46], and in this study, IRW had both high *E. coli* loads and diverse isolates. The high contamination of IRW was attributed to the canals that directly connect to the contamination source (i.e., MWW), thereby transmitting waterborne pathogens to crops in the field. Weller et al. [47] suggested that IRW is a point source for *Listeria* contamination in spinach farms and that rain increases its prevalence through non-point source mechanisms. Although the application of MNR may be another source, some isolates in the mixed clusters were collected from sites without MNR. Moreover, the location (direction and elevation) of both PM and QT from MWW hinders backflow toward the city; hence, the relatedness and shared clustering with MWW confirms that the drainage of MWW is the source of *E. coli* contamination in this study.

The direction and spread of *E. coli* from the Perfume River were inclined toward QT since more isolates were recovered from this site, yet PM is closer to the city. We expected PM to have a higher level of contamination than QT due to its proximity; however, the results showed the opposite. The most probable route of exposure to *E. coli* is through irrigation canals, which connect directly to the Perfume River in QT, while in PM, the IRW is withdrawn from a secondary canal. Most STs in IRW samples showed relatedness to MWW isolates from TK, confirming the Perfume River as the route of *E. coli* dissemination.

The genetic diversity of *E. coli* in the environment fluctuates due to various seasonal and annual environmental factors, and the occurrence and population structure of this species vary seasonally [48]. Shifts in the diversity of *E. coli* by season were also noted from the 5.1% of the total STs shared between the wet and dry seasons. A wide range of *E. coli* strains were detected in this study, with STs that have not been identified elsewhere (Table S2). Some of these strains may be of direct concern to human health, while others may be free-living [20]. Although this study focused on the dispersion of *E. coli* as an indicator of fecal bacteria, neither tested pathogenicity nor antimicrobial resistance, some STs and their associated STcs, which were identified here, have been classified as pathogenic and have been found to harbor antibiotic resistance (e.g., ST 48 and STc 10 and 23) [49] while others are potential carriers (e.g., STc 10, 40, and 155) or disease-causing agents (ST 93 and STc 31) [50,51]. EAEC, UPEC, ExPEC, STEC, and APEC pathologies were associated with our results; moreover, STc 95, a classical ExPEC, is known for over half of NMEC and a predominant APEC causing ST was also found in this study [22]. The dominant phylogroups A and B1 have been implicated in a number of pathotypes; however, there is no clear-cut grouping of certain phylogroups with certain pathotypes [22,23]. Furthermore, the largest mixed cluster in the wet season (MNR, MWW, and IRW isolates) had six isolates with the ST10 complex and was closely related to ST10019 and ST10032 (cluster W4, Figure 2b). Strains with STc 10 were the most detected and widespread, with eight isolates from MWW, five from MNR, and one identified in IRW. The highest rates of mutation and recombination were found in STc 10 [50], which may explain the results of cluster W4. Moreover, STc 10 has also been associated with diarrhea in children ten months and older in Nigeria [52]. However, it should be noted that the association of STs with either disease or antibiotic resistance may vary regionally, as observed by Nadimpalli et al. [53].

Floods directly change the transport of contaminants, can affect existing water and sanitation infrastructure [2,7,15] and may extend into agricultural areas [54,55]. The evidence of infection risk following flood events was well documented by Paterson et al. [16]. The effects of floods may appear weeks to months after a flood event, depending on the routes of transmission and exposure. In this study, *E. coli* was isolated from vegetables grown in peri-urban farms during the dry season, indicating that there is a risk of contamination weeks after the flooding. From the results, irrigation canals are significant routes for *E. coli* dispersion through the direct link to the highly contaminated river which receives urban drainage. The STs in IRW were closely related to those in MWW isolates, indicating wastewater and urban drainage as the source of *E. coli.*

## 5. Conclusions

Although rainfall events reduce pathogen concentrations through dilution, the heightened spread of microbial contaminants during the wet season of the East Asian Monsoon presents great public health risks due to infections caused by the pathogens carried in the stormwater and run-off. In this study, variations in *E. coli* concentrations between the dry and wet seasons were negligible. Despite the low concentrations resulting from dilution, *E. coli* strains with links to MWW were more widespread during the wet season. Moreover, IRW from the Perfume River was highly contaminated with *E. coli*, and evidence of contaminant transfer was also found at low levels in VEG samples. Although our results are based on sampling for only two months per season and two rainfall events, we were able to demonstrate the enhanced spread of *E. coli*—an indicator of fecal contamination—from Hue City to surrounding agricultural areas during the wet season. This enhanced dispersion signifies a substantial bacterial spread that poses a risk to farmers and farm contamination. These findings may improve our understanding of the impacts of MWW discharge and seasonal flooding on the contamination of peri-urban agricultural areas and highlights the pathogenic strains present in the Perfume River. The high diversity in *E. coli* with links to MWW calls for measures to treat and monitor urban drainage before discharge to water bodies. Our results are particularly important for the planning and installation of flood prevention and wastewater treatment infrastructure.

## Figures and Tables

**Table 1:** Primer and the annealing temperature settings for MLST analysis (adapted from [31]).

Target Gene	Primer	Sequence	Product Size	Annealing Temperature
*adk*	adk-F	TCATCATCTGCACTTTCCGC	766 bp	54 °C
	adk-R	CCAGATCAGCGCGAACTTCA		
*fumC*	fumC-F	TCACAGGTCGCCAGCGCTTC	806 bp	65 °C
	fumC-R	GTACGCAGCGAAAAAGATTC		
*gyrB*	gyrB-F	TCGGCGACACGGATGACGGC	815 bp	60 °C
	gyrB-R	GTCCATGTAGGCGTTCAGGG		
*icd*	icd-F	ATGGAAAGTAAAGTAGTTGTTCCGGCACA	878 bp	62 °C
	icd-R	GGACGCAGCAGGATCTGTT		
*mdh*	mdh-F	AGCGCGTTCTGTTCAAATGC	799 bp	56 °C
	mdh-R	CAGGTTCAGAACTCTCTCTGT		
*purA*	purA-F	TCGGTAACGGTGTTGTGCTG	845 bp	58 °C
	purA-R	CATACGGTAAGCCACGCAGA		
*recA*	recA-F	CGCATTCGCTTTACCCTGACC	734 bp	58 °C
	recA-R	TCGTCGAAATCTACGGACCGGA		

**Table 2:** Number of *E. coli* isolates for samples collected during the dry and wet season in 2018.

Sample Type	Number of *E. coli* Isolates	
	Dry Season	Wet Season
Manure (MNR)	-	18
Soil (SOL)	16	11
Irrigation water (IRW)	40	39
Wastewater (MWW)	13	19
Vegetable (VEG)	21	ND
Total	90	87

- No sample; ND—Isolates were not included in MLST analysis.

**Table 3:** Previously reported pathotype and phylogroups associated sequence types in clusters formed from phylogenic analysis.

Season	Cluster	ST	ST Complex	Pathotype ^a^	Phylogroup ^a^ (Clermont)	Phylogroup ^a^ (EzClermont)	Sample
Dry	1	10	STc10	UPEC, STEC, VTEC, NMEC, ExPEC, ETEC, EPEC, EAEC, APEC, Non-pathogen	E or clade I, C, A	U, cryptic, C, A	MWW
	2	409	None	Pathogen (Unknown)	B1 and A	A	MWW, IRW
	3	181	STc168	Unknown, Non-pathogen	A	A	VEG
	4	161	None	Pathogen	A	A	IRW
	5	10,865	None	NA			IRW
	6	10,867	None	NA			VEG
	7	196	None	UPEC, Non-pathogen	B1	B1 and A	SOL
		10,678	None	NA			SOL
	8	10,688	None	NA			IRW
Wet	1	1148	None	Pathogen, Non-pathogen	B1	B1	SOL
	2	1056	None	NA	B1	B1	IRW
	3	10,012	None	NA			MNR
	4	200	STc40	VTEC, EHEC, EAEC, Non-pathogen	B1	B1	MWW
		40		STEC, EPEC, EAEC, Non-pathogen	B1	B1	IRW
	5	10,023	None	NA			IRW
		10,027	None	NA			IRW
	6	641	STc86	ExPEC, Non-pathogen	D, B1, A	U, D, B1, A	MNR
	7	155	STc155	UPEC, STEC, ExPEC, ETEC, EAEC, APEC, Non-pathogen	B1, A	U, B1, A	IRW
	8	10,008	None	NA			MNR
	9	181	STc168	Non-pathogen	A	A	IRW
	10	93	168	UPEC, ExPEC, ETEC, DAEC, APEC, Non-pathogen	E, D, A	D, A	IRW, MWW, MNR
		9872	None	NA			
	11	48	10	UPEC, ExPEC, EAEC, APEC, Non-pathogen	E, A	Cryptic, E, A	MNR, MWW, IRW, SOL
		10,032	None	NA			No Database
		6856	10	NA	A	A	
		10,019	None	NA			
	12	10,033	None	NA			IRW
			Phylogroup				
Summary		Sample	A	B1	B2	E	
		MNR	3	4			
		VEG	5	3			
		SOL	1	9			
		IRW	18	15			
		MWW	14	3	1	1	

Underline—most prevalence, NA—not available, EPEC: Enteropathogenic *E. coli*, ETEC: Enterotoxigenic *E. coli*, EAEC: Enteroaggregative *E. coli*, EHEC: Enterohaemorrhagic *E. coli*, EIEC: Enteroinvasive *E. coli*, ExPEC: Extraintestinal Pathogenic *E. coli*, DAEC: Diffusely Adherent *E. coli,* STEC: Shiga Toxin-producing *E. coli*, UPEC: Uropathogenic *E. coli*, APEC: Avian Pathogenic *E. coli*, VTEC: Verotoxigenic *E. coli*, NMEC: Neonatal Meningitis *E. coli.* ^a^ result from the Enterobase [33].

## Data Availability

Data will be shared on request.

## Supplementary Materials

The following are available online at https://www.mdpi.com/article/10.3390/ijerph18189580/s1, Figure S1: E.coli loads in soil, vegetable, and irrigation water from all the sites throughout the sampling season. The open circles are for soil control samples plotted on the secondary axis, Table S1: Average number of E. coli in the soil, vegetables, irrigation water, manure and municipal wastewater from Huong Chu (HC), Phu Mau (PM), Quang Thanh (QT), and Toa Kham (TK) communes during a year (May 2018 to April 2019), Table S2: Sequence type (ST) and sequence type complex (ST Complex) identity of E. coli isolates collected during the study and their reported phylogroup.
