# Supplementary material for: Tracking Fecal Bacterial Dispersion from Municipal Wastewater to Peri-Urban Farms during Monsoon Rains in Hue City, Vietnam
Source: Int J Environ Res Public Health. 2021 Sep 11;18(18):9580. doi: 10.3390/ijerph18189580 (PMC8468961; doi:10.3390/ijerph18189580)
Supplement: Supplementary file 1 [file ijerph-18-09580-s001.zip › ijerph-1347063-supplementary.pdf]

Supplementary Data

Impact of Municipal Wastewater on *E. coli* Contamination and its Seasonal Change in Peri-Urban Farms of Hue City, Vietnam

Table S1. Average number of *E. coli* in the soil, vegetables, irrigation water, manure and municipal wastewater from Huong Chu (HC), Phu Mau (PM), Quang Thanh (QT) and Toa Kham (TK) communes during a year (May 2018 to April 2019).

**Sample**	**Unit**	**2018**								**2019**			
		**May**	**Jun**	**Jul**	**Aug**	**Sep**	**Oct**	**Nov**	**Dec**	**Jan**	**Feb**	**Mar**	**Apr**
**Huong Chu (HC)**		Dry season				Wet season					Dry season		
Soil	CFU/g	2.5×10^{1}	4.0×10^{1}	1.6×10^{1}	0.0×10^{0}	3.1×10^{1}	0.0×10^{0}	3.3×10^{0}	6.6×10^{0}	2.0×10^{0}	1.5×10^{1}	0.0×10^{0}	0.0×10^{0}
Vegetables	CFU/g	9.8×10^{0}	0.0×10^{0}	2.7×10^{1}	3.3×10^{0}	0.0×10^{0}	1.6×10^{0}	0.0×10^{0}	0.0×10^{0}	0.0×10^{0}	0.0×10^{0}	0.0×10^{0}	0.0×10^{0}
Irrigation Water	CFU/ml	0.0×10^{0}	1.7×10^{0}	5.5×10^{1}	2.0×10^{1}	7.0×10^{0}	1.0×10^{1}	3.5×10^{1}	1.7×10^{1}	1.5×10^{1}	1.8×10^{1}	1.2×10^{1}	1.7×10^{1}
Manure	CFU/g	-	-	-	-	-	-	-	-	-	-	-	-
**Phu Mau (PM)**													
Soil	CFU/g	3.9×10^{1}	1.8×10^{2}	3.0×10^{0}	0.0×10^{0}	3.0×10^{0}	3.9×10^{1}	5.9×10^{1}	4.6×10^{1}	0.0×10^{0}	0.0×10^{0}	0.0×10^{0}	0.0×10^{0}
Vegetables	CFU/g	7.7×10^{-1}	1.4×10^{-1}	1.0×10^{0}	0.0×10^{0}	0.0×10^{0}	0.0×10^{0}	0.0×10^{0}	0.0×10^{0}	0.0×10^{0}	5.0×10^{0}	0.0×10^{0}	0.0×10^{0}
Irrigation Water	CFU/ml	2.9×10^{1}	1.9×10^{1}	3.0×10^{0}	1.7×10^{1}	1.4×10^{1}	3.0×10^{0}	4.0×10^{0}	5.1×10^{0}	2.4×10^{0}	3.2×10^{1}	5.1×10^{0}	4.3×10^{0}
Manure	CFU/g	-	-	-	-	-	-	-	1.7×10^{3}	-	-	-	-
**Quang Thanh (QT)**													
Soil	CFU/g	4.0×10^{1}	1.6×10^{1}	1.3×10^{1}	0.0×10^{0}	3.0×10^{0}	8.8×10^{1}	1.5×10^{1}	3.7×10^{1}	0.0×10^{0}	2.9×10^{2}	1.7×10^{3}	2.8×10^{2}
Vegetables	CFU/g	1.7×10^{0}	4.6×10^{-1}	1.2×10^{0}	4.2×10^{-1}	3.0×10^{-1}	0.0×10^{0}	1.3×10^{2}	3.8×10^{0}	1.0×10^{-1}	3.5×10^{-1}	1.4×10^{0}	1.2×10^{0}
Irrigation Water	CFU/ml	9.8×10^{1}	6.7×10^{0}	2.5×10^{1}	7.1×10^{0}	1.9×10^{1}	9.3×10^{0}	7.7×10^{0}	6.9×10^{0}	1.1×10^{1}	8.2×10^{0}	7.3×10^{1}	7.0×10^{1}

Manure	CFU/g	-	-	-	-	-	-	-	1.2×10^{1}	-	-	-	-
**Toa Kham (TK)**													
Wastewater	CFU/ml	-	4.0×10^{4}	-	-	-	-	-	1.3×10^{3}	-	-	-	-

Note: - = no samples

Table S2: Sequence Type (ST) and Sequence Type Complex (ST Complex) identity of *E. coli* isolates collected during the study and their reported phylogroup

Dry Season				Wet Season			
**Isolate**	**ST**	**ST Complex**	**Phylogroup**	**Isolate**	**ST**	**ST Complex**	**Reported Phylogroup**
SOL-1 HC4	939	None	**A**	MNR-1 QT1	4681	469	**B1**
SOL-1 QT2	196	None	**B1**	MNR-10 PM5	10017	None	-
SOL-1 QT3	10862	None	-	MNR-2 PM5	9872	None	-
SOL-3 QT2	196	None	**B1**	MNR-2 QT1	10012	None	-
SOL-6 PMc	3640	None	**B1**	MNR-3 PM5	641	86	D, **B1**, A
SOL-1 HC1-1	10355	None	-	MNR-3 QT1	10013	None	-
SOL-1 HC4	10356	None	-	MNR-4 PM5	10008	None	-
SOL-1 PM2	10357	None	-	MNR-4 QT1	10026	None	-
SOL-1 PMc	10677	None	-	MNR-5 PM5	10008	None	-
SOL-2 HC4	10358	None	-	MNR-5 QT1	48	10	E, **A**
SOL-2 QT2	10678	None	-	MNR-6 PM5	10014	None	-
SOL-3 HC1-1	10679	None	-	MNR-6 QT1	10012	None	-
SOL-3 HC4	10359	None	-	MNR-7 PM5	48	10	E, **A**
SOL-3 PMc	10360	None	-	MNR-7 QT1	10015	None	-
SOL-4 HC4	10362	None	-	MNR-8 PM5	10016	None	-
SOL-4 PMc	10361	None	-	MNR-8 QT1	48	10	E, **A**
VEG-1 PM1	9967	446	**B1**	MNR-9 PM5	641	86	D, **B1**, A
VEG-1 QT1	10363	None	-	MNR-9 QT1	48	10	E, **A**
VEG-1 QT4	10681	None	-	SOL-1 PM2	10018	None	-
VEG-1 QT5	10364	None	-	SOL-1 QT1	10019	None	-
VEG-11 HC5	5044	None	**B1**, A	SOL-10 PM2	1148	None	**B1**
VEG-13 HC5	10867	None	-	SOL-2 QT1	10021	None	-
VEG-14 HC5	533	40	**B1**	SOL-3 PM2	1148	None	**B1**
VEG-15 HC5	181	168	**A**	SOL-3 QT1	10020	None	-
VEG-16 HC5	10867	None	-	SOL-4 PM2	1148	None	**B1**
VEG-2 HC5	8369	None	**B1**	SOL-5 HC3	10022	None	-
VEG-2 PM1	10365	None	-	SOL-5 PM2	1148	None	**B1**
VEG-2 PM1	10863	None	-	SOL-6 PM2	1148	None	**B1**
VEG-2 QT5	10680	None	-	SOL-7 PM2	4088	None	**B1**
VEG-3 HC5	10864	None	-	IRW-1 HC	175	None	**A**
VEG-3 QT1	10866	None	-	IRW-1 PM2	10023	None	-
VEG-3 QT5	10682	None	-	IRW-1 PM3	533	40	**B1**
VEG-4 HC5	5229	101	**B1**	IRW-1 QT3+5	10024	None	-
VEG-5 HC5	10867	None	-	IRW-1 QT4	10025	None	-
VEG-5 QT1	10868	None	-	IRW-10 HC	181	168	**A**
VEG-7 HC5	542	None	**A**	IRW-10 QT3+5	10033	None	-
VEG-8 HC5	181	168	**A**	IRW-11 HC	10034	None	-
IRW-1 HC4+5	2522	None	**B1**	IRW-12 QT3+5	165	165	D, **A**
IRW-1 PM2	10687	None	-	IRW-13 QT4	711	None	**B1**
IRW-1 PM3	10366	None	-	IRW-14 QT3+5	101	101	D, **B1**, A

IRW-1 QT1	10869	None	-	IRW-14 QT4	40	40	**B1**
IRW-1 QT2	10683	None	-	IRW-16 QT3+5	394	394	E or clade I, **D**, A
IRW-1 QT2	161	None	**A**	IRW-19 QT3+5	4577	None	**B1**
IRW-1 QT3	10367	None	-	IRW-2 HC	7366	None	**A**
IRW-1 QT4	409	None	B1 and **A**	IRW-2 PM2	10027	None	-
IRW-10 HC4+5	10880	None	-	IRW-2 QT2	401	None	**A**
IRW-11 HC4+5	10878	None	-	IRW-2 QT4	10028	None	-
IRW-12 HC4+5	10881	None	-	IRW-20 QT3+5	155	155	**B1**, A
IRW-13 HC4+5	3106	155	-	IRW-3 PM2	10027	None	-
IRW-14 HC4+5	1727	446	**B1**	IRW-3 PM3	93	168	E, D, **A**
IRW-17 HC4+5	10882	None	-	IRW-3 QT2	6836	165	**A**
IRW-18 HC4+5	10879	None	-	IRW-3 QT4	10029	None	-
IRW-19 HC4+5	58	155	**B1**,A	IRW-4 HC	10030	None	-
IRW-2 HC1	1656	None	**B1**, A	IRW-4 PM2	1056	None	**B1**
IRW-2 PM2	10688	None	-	IRW-5 HC	155	155	**B1**, A
IRW-2 PM3	202	None	**A**	IRW-5 PM2	1056	None	**B1**
IRW-2 QT1	10870	None	-	IRW-5 QT4	10031	None	-
IRW-2 QT2	161	None	**A**	IRW-6 PM2	10027	None	-
IRW-2 QT3	10684	None	-	IRW-6 QT3+5	196	None	**B1**
IRW-3 PM2	10688	None	-	IRW-6 QT4	6856	10	**A**
IRW-3 QT1	10865	None	-	IRW-7 HC	181	168	**A**
IRW-3 QT2	161	None	**A**	IRW-7 PM2	10027	None	-
IRW-3 QT3	10368	None	-	IRW-7 QT4	10032	None	-
IRW-4 PM2	10685	None	-	IRW-8 HC	181	168	**A**
IRW-4 QT1	10871	None	-	IRW-8 PM2	10027	None	-
IRW-4 QT3	10369	None	-	IRW-8 QT3+5	10033	None	-
IRW-5 HC4+5	10872	None	-	IRW-9 PM2	6109	None	**B1**
IRW-5 PM2	10689	None	-	IRW-9 QT4	5229	101	**B1**
IRW-5 QT1	10873	None	-	MWW-1	205	205	**B1**
IRW-5 QT3	10370	None	-	MWW-10	10037	None	-
IRW-6 HC4+5	10874	None	-	MWW-12	48	10	E, **A**
IRW-6 QT1	10865	None	-	MWW-13	10038	None	-
IRW-6 QT3	10371	None	-	MWW-15	200	40	**B1**
IRW-7 HC4+5	10875	None	-	MWW-16	3202	None	**A**
IRW-7 QT3	10372	None	-	MWW-18	3240	None	-
IRW-8 HC4+5	10876	None	-	MWW-20	1139	None	**A**
IRW-9 HC4+5	10877	None	-	MWW-21	746	10	**A**
MWW-1 TK	10686	None	-	MWW-22	10039	None	-
MWW-10 TK	10	10	E or clade I, C, **A**	MWW-23	10040	None	-
MWW-11 TK	409	None	B1 and **A**	MWW-27	3856	None	**A**
MWW-12 TK	10	10	E or clade I, C, **A**	MWW-28	218	10	E, **A**
MWW-13 TK	641	86	D, **B1**, A	MWW-29	10041	None	-
MWW-14 TK	10379	None	-	MWW-3	130	31	**E**
MWW-15 TK	218	10	E, **A**	MWW-4	10035	None	-
MWW-2 TK	10	10	E or clade I, C, **A**	MWW-6	95	95	**B2**, B1

MWW-3 TK	10375	None	-	MWW-7	10036	None	-
MWW-4 TK	3489	10	**A**	MWW-9	93	168	E, D, **A**
MWW-5 TK	10691	None	-				
MWW-6 TK	10376	None	-				
MWW-9 TK	93	168	E, D, **A**				

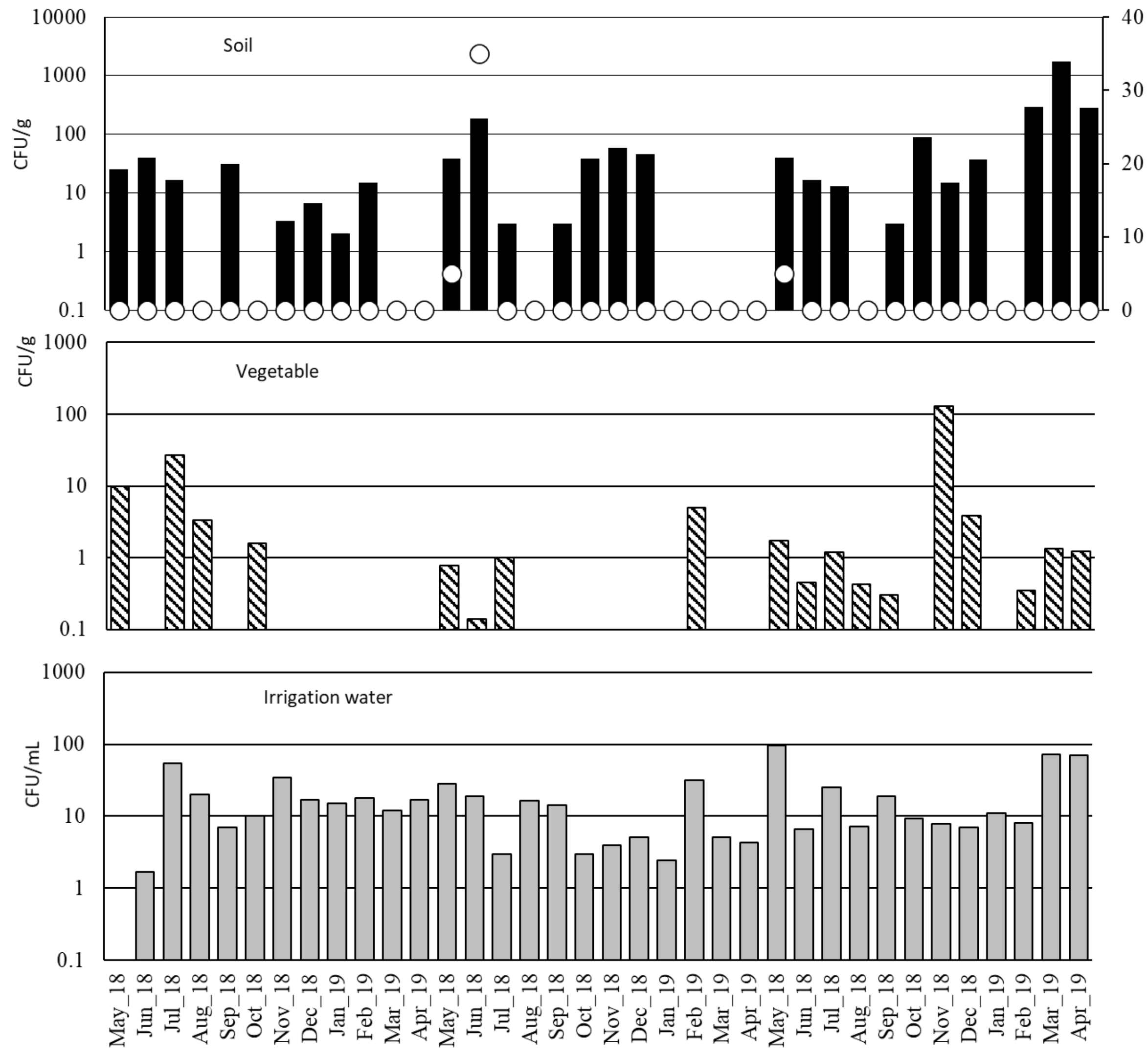

Figure S1. *E.coli* loads in soil, vegetable and irrigation water from all the sites throughout the sampling season. The open circles are for soil control samples plotted on the secondary axis
